# Feasibility Study on Quantification of Biodegradable Polyester Microplastics Based on Intrinsic Fluorescence

**DOI:** 10.3390/polym17212953

**Published:** 2025-11-05

**Authors:** Tian-Chao Shi, Ze-Yang Zhang, Xiao-Han Zhou, Xing Zhang, Shao-Chuang Su, Hong Yang, Hao-Bo Chai, Ge-Xia Wang, Jun-Hui Ji, Yue Ding, Xu-Ran Liu, Dan Huang

**Affiliations:** 1 College of Materials Engineering, North China Institute of Aerospace Engineering, Langfang 065000, China; stc18330656926@163.com (T.-C.S.); zhouxiaohanna@163.com (X.-H.Z.); xingzhang202506@163.com (X.Z.); su19862096041@163.com (S.-C.S.); yh1145299902@163.com (H.Y.); 18833603477@163.com (H.-B.C.); 2 National Engineering Research Center of Engineering and Ecological Plastics, Technical Institute of Physics and Chemistry, Chinese Academy of Sciences, Beijing 100190, China; zhangzeyang21@mails.ucas.ac.cn (Z.-Y.Z.); gxwang@mail.ipc.ac.cn (G.-X.W.); jhji@mail.ipc.ac.cn (J.-H.J.); 3 School of Material and Chemical Engineering, Xuzhou University of Technology, Xuzhou 221018, China

**Keywords:** biodegradable microplastics, PBAT, fluorescence quantification, visualization, rapid detection

## Abstract

While biodegradable plastics alleviate plastic pollution, their degradation-derived biodegradable microplastics (BMPs) pose new ecological risks, necessitating efficient quantification methods. This study explores a label-free approach by leveraging the intrinsic fluorescence of common biodegradable polyesters (PLA, PHB, PBS, PBAT, PCL). We find that biodegradable microplastics exhibit two types of characteristic fluorescence emission: one originating from molecular functional groups and the other originating from the chromophore formed by the aggregation of conjugated groups. Using PBAT as a model, we confirm that fluorescence intensity depends on the BMPs’ size and shape. Under 380 nm excitation, concentration-dependent signals are observed at 436 nm (indirectly from PBAT-enhanced water Raman scattering) and 465 nm (directly from PBAT intrinsic fluorescence), leading to successful linear models between BMPs’ mass concentration and fluorescence intensity over 100–500 mg/L, with correlation coefficients (R^2^) of 0.877 and 0.963, respectively. Compared with the fluorescence labeling method, the intrinsic fluorescence approach achieves comparable R^2^ while exhibiting lower signal intensity (~10^3^). Nevertheless, its operational simplicity offers a distinct advantage for the rapid quantification of pre-isolated and purified microplastics.

## 1. Introduction

As a new type of pollutant, ‘microplastics’ (<5 mm) have received widespread global attention in recent years [1]. They are widely distributed in the soil and ocean and will not disappear for hundreds of years due to their excellent weather resistance. MPs may pose a serious threat to natural organisms and even the entire ecosystem due to their chemical properties, additives, and the fact that they can be used as carriers of toxic substances or pathogens [2,3,4,5,6,7,8,9]. They can even enter the food chain through air, drinking water, salt, animals and other media, thus affecting human health [10,11,12]. Biodegradable plastics are one of the most important ways to solve plastic pollution at the source. Biodegradable plastics, mainly polylactic acid (PLA), polyhydroxyalkanoates (PHAs), polybutylene adipate/terephthalate (PBAT), polybutylene succinate (PBS), and polycaprolactone (PCL), are becoming increasingly popular. However, more and more studies have shown that compared with traditional durable plastics, biodegradable plastics are more likely to disintegrate during the degradation process, resulting in the concentrated release of more random particles or fragments similar in size to microplastics in a short period of time [13,14,15,16], which is likely to have a more negative impact on the ecosystem [17,18,19,20,21,22,23,24]. Fritz et al. revealed a 20% to 50% reduction in the biomass of garden cress, wheat, and rapeseed when grown in soil amended with 2% biodegradable plastic mulch fragments [25]. Shenkar et al. demonstrated that PLA microplastics could adversely affect the fertilization success of the marine organism *Microcosmus exasperates* [26]. It can be seen that BMPs have become an emerging environmental pollutant, and their accurate detection is a key prerequisite for assessing their environmental occurrence status and ecological risks.

Most existing BMPs detection methods follow the traditional microplastic detection systems, which mainly include the visual method, spectroscopy and mass spectrometry. The visual method usually uses a microscope to sort, identify and count microplastics. It is a relatively simple and intuitive method that is suitable for microplastics ranging from a few hundred micrometers to millimeters [27,28,29]. However, this method is less effective when detecting small-sized microplastics or other similar interferences, and the error increases as the particle size decreases. The chemical composition of microplastics can be analyzed with the help of characteristic spectra of spectroscopy (Fourier transform infrared spectroscopy (FTIR), Raman spectroscopy and laser direct infrared (LDIR)) [30,31,32,33,34,35]. However, due to local changes in the chemical structure, the matching degree between the measured spectrum and the standard spectrum library will decrease, which will significantly increase the identification error rate and is very labor-intensive. Mass spectrometry mainly includes pyrolysis-gas chromatography/mass spectrometry (Pyr-GC/MS), thermal extraction–thermal desorption gas chromatography/mass spectrometry (TED-GC/MS), and liquid chromatography/mass spectrometry (LC–MS), etc. [36,37]. In the pyrolysis method, different microplastics will produce different characteristic pyrolysis products during pyrolysis. By establishing a database of different microplastic mass-characteristic pyrolysis product calibration curves, different microplastics in the environment can be quantitatively analyzed [38,39,40,41]. However, it is difficult to distinguish complexes with similar mass and degradation temperature data, and destructive sampling and high equipment requirements are required, making it difficult to routinely apply. LC–MS requires the use of specific solvents to depolymerize microplastics into monomers or small molecular compounds before analysis [42,43]. It is suitable for single component microplastics, but not for materials with complex components and similar structures. In summary, the shortcomings of the current methods for detecting BMPs are as follows: The visual method has poor effect and large error on small-sized or similar interferences; spectroscopy is susceptible to chemical structure changes, resulting in a decrease in matching and is time-consuming; mass spectrometry has problems such as destructive sampling, high equipment requirements, and difficulty in distinguishing similar compounds or complex components. Therefore, it is of great significance to develop a simple, rapid and accurate method for quantitative determination of biodegradable microplastics.

Fluorescence quantification is a highly sensitive and visual detection method that can quantify microplastics through fluorescence intensity, greatly shortening the detection time [44,45]. Currently, the main method used to visualize the migration and content change of microplastics is fluorescence labeling (fluorescent dyes or probe molecules), which monitors changes in absorption or fluorescence [46,47]. However, this method is very specific and only suitable for the detection of mass concentration of fewer kinds of microplastics, and the labeled fluorescence is unstable. H. A. Nel [48] and J. C. Prata [49] found that after staining microplastics with Nile red, the detection rates of various microplastics such as polyethylene (PE) and polypropylene (PP) were higher, but polyethylene terephthalate (PET) and polyvinyl chloride (PVC) could not be accurately evaluated. Moreover, the fluorescence loss of microplastics after staining reached 73.5% within two months, so the samples need to be tested as soon as possible in a short time. Based on this, we hope that the fluorescence quantitative method can achieve long-term stable fluorescence tracking based on the intrinsic fluorescence performance of the material.

In recent years, the proposal of the aggregation-induced emission (AIE) mechanism has led to the emergence of numerous non-traditional fluorescent polymers. These polymers often contain electron-rich chromophores such as benzene rings, ester groups, carbonyls, amides and double bonds. This development has established the feasibility of using intrinsic fluorescence for microplastic tracking, showing significant potential. Preliminary explorations of this fluorescence-based method for identifying conventional microplastics have yielded promising results. For instance, the work by C. M. Penso et al. successfully identified the optimal wavelengths for accurately discriminating six conventional polymers—polyamide 6 (PA6), polymethyl methacrylate (PMMA), PP, polystyrene (PS), high-density polyethylene (HDPE), and PET—by analyzing their fluorescence data under various excitation wavelengths from 245 nm to 345 nm at 10 nm intervals [50]. However, a quantitative model correlating fluorescence signals with microplastic content is still lacking.

To date, there are few studies on the intrinsic fluorescence of biodegradable polyesters [51]. Considering that the molecular chain of biodegradable polyester contains electron-rich ester groups and benzene rings, it will not only produce characteristic peaks due to the conjugated group itself but also may form new chromophores due to the three-dimensional spatial aggregation of conjugated groups in the molecular chain, thereby inducing the red shift of fluorescence characteristic peaks through AIE mechanism [52,53,54]. In our previous work, it was found that the fluorescence intensity of PBAT suspension solution containing microplastics changed significantly with the degradation process, which confirmed the feasibility of monitoring PBAT microplastics by the intrinsic fluorescence method, with the characteristics of visualization and high sensitivity [52].

This study systematically investigated the feasibility of tracking biodegradable polyester microplastics via their intrinsic fluorescence. We began by examining the fluorescence performance of representative biodegradable polyesters, including PLA, poly(3-hydroxybutyrate-co-4-hydroxybutyrate) (P(3HB-co-4HB)), PBAT, PBS, and PCL, with a focus on how particle size and shape influence their fluorescent characteristics. Furthermore, the fluorescence-concentration correlation model of PBAT microplastic suspension was developed. The accuracy of this intrinsic fluorescence-based method was evaluated by comparing it with labeled fluorescence. In addition, the effects of complex environmental matrices such as seawater and soil on the relationship model were also evaluated. The successful implementation of this work lays the foundation for tracking biodegradable microplastics.

## 2. Materials and Methods

### 2.1. Materials

The PLA pellets were Luminy series polylactic acid resin purchased from TotalEnergies Corbion, The Netherlands, and the PHB pellets (P(3HB-co-4HB)) were Phamily series PHB resin obtained from Micro-structured Biotechnology Co., Ltd., Beijing, China. PBAT pellets were obtained from Yihua Chemical Industry Co., Ltd., Hubei, China. PBS pellets were purchased from Lanshan Tunhe Chemical Industry Co., Ltd., Xinjiang, China. PCL pellets were obtained from Ouke New Materials Technology Co., Ltd., Wuhan, China. PET pellets were obtained from China Resources Chemical Materials Technology Co., Ltd., Zhuhai, China. 50-μm PBAT microspheres with a known concentration of microspheres in water (10 mg/mL) were prepared by sending the PBAT pellets to Zhichuan Technology Co., Ltd., Jiangsu, China. Ultrapure water was obtained by self-production of ultrapure water using an ultrapure water preparation system (UPDRO Environmental Protection Equipment Engineering Co., Ltd., Chengdu, China, model: UPDROEMB-500). 6-Aminofluorescein (AF) (>97%) and 1-(3-dimethylaminopropyl)-3-ethyl carbodiimide hydrochloride (EDC, 98%) were purchased from McLean Biochemical Technology Co., Ltd., Shanghai, China. N-Hydroxysuccinimide (NHS, 99%) was purchased from Acmec Biochemical Co. Ltd., Shanghai, China. Dimethyl sulfoxide (DMSO, AR) was obtained from Modern Oriental Technology Development Co., Ltd., Beijing, China. Disodium hydrogen phosphate–sodium dihydrogen phosphate buffer (0.2 mol/L, pH 5.5) was purchased from Boao Tuoda Technology Co., Ltd., Beijing, China. The property parameters of biodegradable polyester raw materials are shown in Table 1. Filtered seawater was obtained by filtering the Bohai Sea water with 0.45 μm filter membrane. Saturated ZnCl_2_ solution and the filtrate obtained from soil flotation using saturated ZnCl_2_ solution were self-made.

### 2.2. Preparation of Polyester Microplastics

#### 2.2.1. Preparation of Random Microplastics

Different raw material pellets or films were placed in a beaker, quenched with liquid nitrogen and then quickly crushed. Microplastics with sizes in a mesh range of 20–40, 40–140 and 140–300 were screened out using sieves of 20 mesh (pore size 900 μm), 40 mesh (pore size 400 μm), 140 mesh (pore size 100 μm) and 300 mesh (pore size 50 μm). The raw material pellets were prepared into particle microplastics, and the sample films were prepared into film microplastics. The PBAT film was obtained by casting the raw material pellets at a casting temperature of 150 °C, and the thickness was about 100 μm.

#### 2.2.2. Preparation of Fluorescently Labeled Microplastics

First, the fluorescein solution of AF was prepared by dissolving AF in DMSO at a concentration of 10 mmol/L (0.01 mol/L). Then, the AF stock solution was diluted to 15.6 μM using 1.0 mmol/L EDC, 1.0 mmol/L NHS, and a buffer solution (pH = 5.5). Subsequently, the microplastics were immersed directly into the prepared AF fluorescent solution and sonicated for 15 min. Finally, they were washed with ethanol to remove any excess AF fluorescent solution. After the labelling process, the film was dried for further use and analysis.

### 2.3. Preparation of PBAT Microplastic Suspension

Using ultrapure water as a solvent, 1 mg, 2 mg, 4 mg, 6 mg, 8 mg and 10 mg PBAT micro plastics with particle size of 140–300 mesh were accurately weighed and added to 20 mL ultrapure water, and sonicated in an ultrasonic cleaner for 5 min to prepare 50 mg/L, 100 mg/L, 200 mg/L, 300 mg/L, 400 mg/L, 500 mg/L suspensions. The 50 mg/L suspension was further diluted into suspensions with concentrations of 10 mg/L, 20 mg/L, 30 mg/L, and 40 mg/L, respectively. According to the above process, 10 mg/L, 20 mg/L, 30 mg/L, 40 mg/L, 50 mg/L, 100 mg/L, 200 mg/L, 300 mg/L, 400 mg/L and 500 mg/L suspensions of 50-μm PBAT microspheres and fluorescently labeled PBAT microplastics with particle size of 140–300 mesh were also prepared.

### 2.4. Characterization Analysis

1. Molecular weight test: 1515 gel permeation chromatography (GPC) of Waters Ltd. in USA was used to test the molecular weight and distribution of PLA, P(3HB-co-4HB), PBAT, PBS and PCL. The mobile phase was chloroform (CHCl_3_), and the flow rate was 1 mL/min, and the standard sample was PS. The molecular weight and distribution of PET were tested with hexafluoroisopropanol (HFIP) as the mobile phase, the flow rate was 1 mL/min, and the standard sample was PS.
2. Film thickness test: The thickness of film was measured by micrometer, and the average value of the thickness of five places was taken.
3. Particle size test: The particle size of microplastics was photographed by JCM-6000 scanning electron microscope of JEOL Ltd. in Japan and the particle size distribution was obtained by particle size analysis software Nano Measurer (version 1.2.5).
4. Fluorescence images: Different microplastics were fluorescently irradiated and photographed using an ultraviolet lamp with a wavelength of 365 nm in a ZF-203C dark box three-purpose ultraviolet analyzer of Xiuilab Instrument Co., Ltd. in Shanghai, China.
5. Three-dimensional fluorescence test: Six polyester powders were subjected to three-dimensional wavelength fluorescence scanning using a F7000 fluorescence spectrophotometer of Hitachi Ltd. in Japan with an excitation voltage of 700 V, Ex: 200–600 nm/Em: 200–750 nm. The excitation wavelength interval was 10 nm, the emission wavelength interval was 5 nm, the scanning speed was 2400 nm/min, and the excitation/emission slit width was 5 nm.
6. Fluorescence spectroscopy test: Different polyester microplastics and their suspensions were measured by a F-4500 fluorescence spectrophotometer of Hitachi Ltd. in Japan. The excitation and emission slit widths were 3 nm, the scanning speed was 5 nm s^−1^, and the voltage was 220 V.

## 3. Results and Discussion

### 3.1. The Intrinsic Fluorescence of Biodegradable Polyester Microplastics

**Figure 1:**
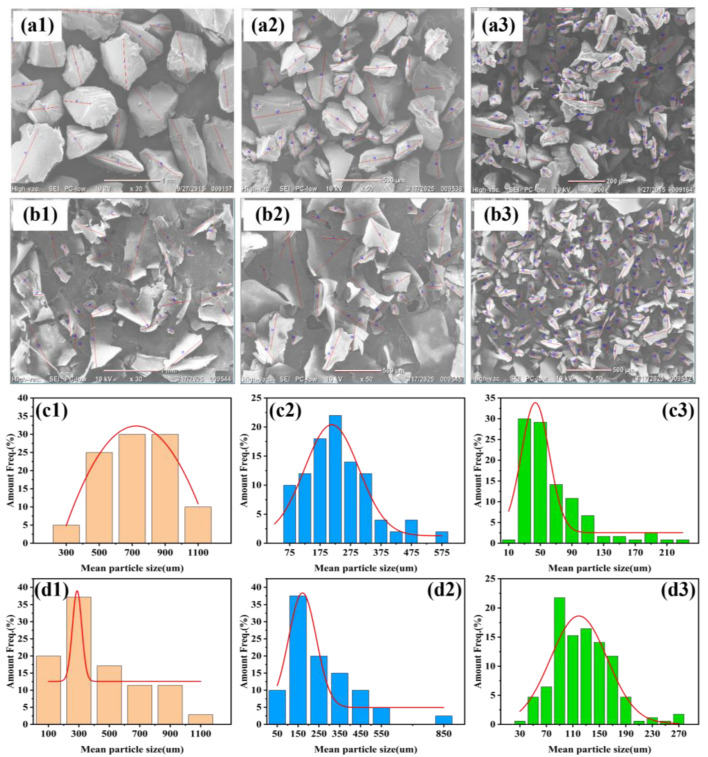
SEM distribution of biodegradable polyester PBAT microplastics: (**a**) PBAT (from pellets); (**b**) PBAT (from film); and particle size distribution; (**c**) PBAT (from pellets); (**d**) PBAT (from film); Numbers 1–3 represent microplastics with a mesh range of 20–40, 40–140 and 140–300.

To simulate the fragments or microparticles formed by random disintegration due to local stress changes during the biodegradation process, low-temperature crushing was used to prepare biodegradable polyester microplastics [55]. After sieving through sieves with different mesh sizes, microplastics with sizes ranging from 20–40 mesh, 40–140 mesh, and 140–300 mesh were obtained (Figure 1, Figures S1 and S2). It can be seen that through the direct quenching and crushing process, the raw material pellets and films were formed into particles and film (fragments) microplastics, respectively, and both showed irregular shapes [56]. Comparing the sizes of microplastics (Table 2), in the 20–40 mesh range, PET particles were relatively large, with the average particle size increasing by about 10%; while in the smaller size ranges of 40–140 and 140–300 mesh, PLA particles were relatively large, with the average particle size even about twice that of other polyester particles, indicating that it is more difficult to further crush into smaller particles. For the same polyester PBAT, the average particle size of its microplastic particles and films is relatively close in the 40–140 mesh range, and there are large differences in the other two size ranges. The preparation methods yielded three distinct batches of random polyester microplastics with varied and well-defined size distributions, which closely mimic the random fragmentation that occurs during biodegradation.

**Figure 2:**
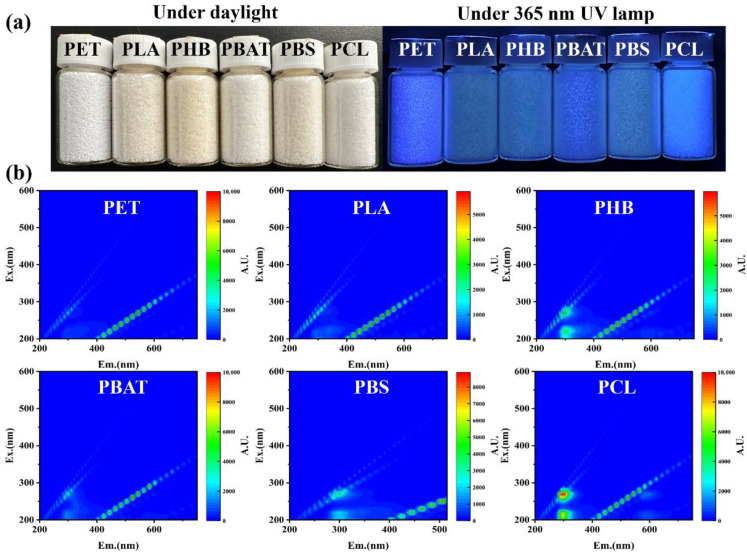
PET and different biodegradable polyester microplastic particles: (**a**) photos under sunlight and 365 nm ultraviolet lamp (particle with a mesh range of 20–40); (**b**) the three-dimensional fluorescence spectrum (powder).

In order to study the fluorescence performance of different biodegradable polyester microplastics, the obtained microplastics were compacted and irradiated with the most commonly used wavelength of 365 nm by the ultraviolet lamp (as shown in Figure 2a). It can be seen that compared with sunlight, all polyester microplastic particles showed more significant differences under 365 nm ultraviolet irradiation, that is, different degrees of fluorescence emission. PET microplastics showed blue-purple fluorescence, while five biodegradable polyester microplastics showed relatively stronger or weaker fluorescence. At the same time, PLA, PHB and PBS showed low autofluorescence in the same particle size range (Figures S3 and S4), which is consistent with the literature report [52,57,58]. In contrast, PBAT and PCL showed stronger autofluorescence, which may be due to the presence of benzene rings, or that the polyester molecular chain is more flexible, resulting in enhanced aggregation of conjugated groups.

To systematically evaluate the fluorescent performance of different polyester materials, a full-wavelength fluorescence scan was performed on all biodegradable polyesters in powder form (Figure 2b). The results reveal that all polyesters exhibit intrinsic fluorescence at the emission wavelength of around 300 nm, attributable to their conjugated groups such as ester groups and benzene rings in the molecular chain [59]. Concurrently, the AIE effect induces varying degrees of red shift in each polyester, resulting in distinct characteristic fluorescence peaks. The differential spectral profiles obtained under various excitation wavelengths thus enable their discrimination through spectral analysis. It is noteworthy that this fundamental principle of discrimination via intrinsic fluorescence is equally applicable to conventional plastics. As reported in the work by C. M. Penso et al., a study on various traditional polymer films also achieved accurate differentiation, with the strongest emission wavelengths ordered as follows: PA6 (400–430 nm), PMMA (350–400 nm), HDPE (~350 nm), PET (380–400 nm), PS (~285 nm), and PP (~270 nm) [50]. In conclusion, the findings from our study on biodegradable polyesters and the documented evidence from traditional plastics jointly demonstrate that the intrinsic fluorescence-based analytical strategy possesses broad applicability, enabling the effective discrimination and identification of most microplastics. It is worth noting that the fluorescence of different sample forms (such as microplastics, powders and standard tensile splines) is different [50,51,52,58,60]. PET powder in our work excited at 200–250 nm has a strong emission at 280–380 nm, contrasting with PET film, which shows strong emission at 380–400 nm when excited at 330–350 nm [50].

**Figure 3:**
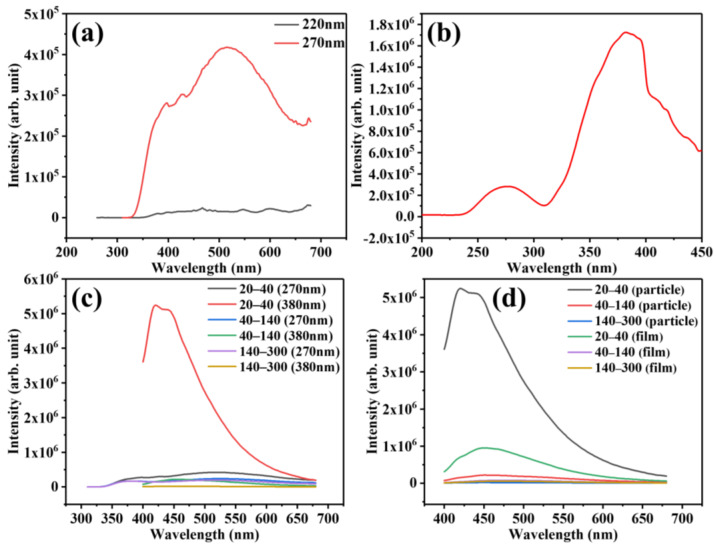
Fluorescence spectra of PBAT particles microplastics: (**a**) Fluorescence emission spectra of PBAT particles microplastics (particle size between 20–40 mesh) excited by 220 nm and 270 nm wavelengths and (**b**) Fluorescence excitation spectra with emission wavelength of 510 nm; (**c**) Fluorescence emission spectra of PBAT particle microplastic at 270 nm and 380 nm excitation wavelengths; (**d**) Fluorescence emission spectra of PBAT particle and film microplastic at 380 nm excitation wavelength.

Due to the good intrinsic fluorescence, PBAT microplastics were selected for in-depth fluorescence analysis, which was also proved in our previous work [52]. From the three-dimensional fluorescence spectrum (Figure 2b), it can be obtained that PBAT has two strong fluorescence emissions, and the excitation and emission wavelengths are 220 nm/295 nm and 270 nm/510 nm, respectively. Then, PBAT (particle size of 20–40 mesh) was excited by ultraviolet light with 220 nm and 270 nm as excitation wavelengths, respectively. As shown in Figure 3a, it can be clearly observed that PBAT has stronger fluorescence emission when excited at 270 nm wavelength, and the emission wavelength is about 510 nm. In order to further explore the fluorescence of PBAT, we obtained the fluorescence excitation spectrum of PBAT at 510 nm in the visible range (Figure 3b). The results reveal two distinct excitation maxima at 270 nm and 380 nm, with significantly enhanced fluorescence intensity at 380 nm attributable to aggregation-induced emission (AIE) effect. In the same size of PBAT particles and film microplastics, the fluorescence intensity at the two excitation wavelengths (Figure 3c and Figure S5a) was compared, and the same conclusion was obtained. Therefore, 380 nm is selected as the most suitable excitation wavelength for PBAT for subsequent fluorescence research.

In order to investigate the effect of size difference on the fluorescence of polyester microplastics, firstly, all polyester microplastic particles were photographed under fluorescence irradiation and the corresponding fluorescence spectra were obtained under the excitation of a unified 365 nm wavelength (Figures S3 and S4). The results show that under fluorescence irradiation, the fluorescence emission of all polyester microplastics decreases with the decrease of particle size, which is reflected in the decrease of fluorescence intensity in the fluorescence spectrum, accompanied by a slight red shift. Furthermore, in PBAT particles microplastics with different size ranges, under the excitation of 270 nm and 380 nm wavelengths (Figure 3c), it is also obtained that under the same wavelength excitation, the fluorescence intensity decreases significantly with the decrease of size. The same fluorescence intensity changes are also obtained in PBAT film microplastics of different sizes (Figure S5a,b). The above results may be mainly due to the large specific surface area of small-sized polyester microplastics, which exposes more defects and dissipates energy through non-radiative transitions, resulting in a decrease in fluorescence intensity.

In order to investigate the effect of shape difference on the fluorescence of polyester microplastics, the fluorescence characteristics of PBAT particles and film microplastics under 380 nm excitation were compared. As shown in Figure 3d, compared with PBAT particle microplastics, the fluorescence emission wavelength of film microplastics is relatively more red-shifted, but the fluorescence intensity is significantly reduced. The same results are obtained under the excitation of 365 nm wavelength (Figure S5c). Considering the larger exposure area of the film, it may also be due to the exposure of more surface defects, which becomes a non-radiative recombination center, consumes excited state energy, and thus leads to a decrease in fluorescence intensity. In summary, the structural differences between two-dimensional films and three-dimensional particles, as well as the differences in size, will have a non-negligible impact on fluorescence characteristics.

### 3.2. Feasibility Analysis of Tracking Microplastics Based on Intrinsic Fluorescence

#### 3.2.1. Construction of Microplastic Fluorescence Correlation Model

Based on the above research results, in order to explore the relationship between microplastics concentration and fluorescence characteristics, PBAT particles microplastics (particle size of 140–300 mesh) were selected for suspension preparation, and then the fluorescence characteristic peaks at 380 nm excitation wavelength were measured. It should be emphasized that in order to reduce the influence of PBAT hydrolysis on fluorescence, the fluorescence measurements were conducted on freshly prepared suspensions shortly after dispersion, and the overall scanning time was about 50 s (400–650 nm).

**Figure 4:**
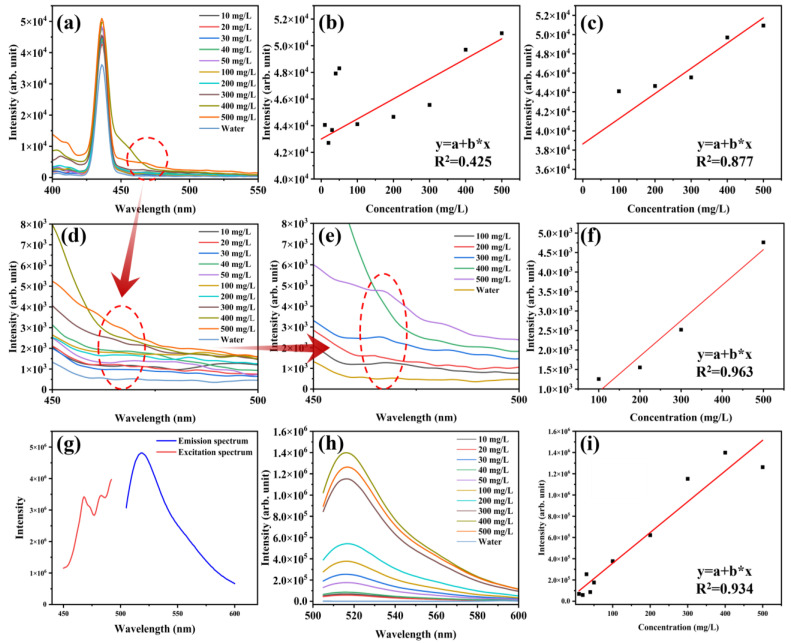
Fluorescence spectra of different concentrations of PBAT particles microplastic suspensions: (**a**) under excitation at 380 nm and (**h**) fluorescently labeled PBAT particles microplastics under excitation at 492 nm; (**b**,**c**) are the mass concentration–fluorescence intensity linear fitting curve of (**a**) at 436 nm; (**d**,**e**) are the enlarged images at 450–500 nm in (**a**); (**f**) is the mass concentration–fluorescence intensity linear fitting curve of (**a**) at 456 nm; (**g**) Fluorescent excitation and emission spectra of AF fluorescent solution; (**i**) is the mass concentration–fluorescence intensity linear fitting curve of (**h**) at 518 nm.

As shown in Figure 4a, a narrow characteristic peak at 436 nm is observed in all PBAT microplastic suspensions, consistent with the ultrapure water blank. The intensity of this peak gradually increases with higher microplastic concentrations. Concurrently, the intrinsic fluorescence characteristic of PBAT microplastics—a broad peak around 465 nm—progressively emerges and intensifies (Figure 4d,e). The enhancement of both characteristic peaks is directly or indirectly induced by the increased presence of PBAT microplastics: (1) The peak at 436 nm can be attributed to the Raman scattering peak of water. The scattering effect of the microplastics enhances the interaction between the excitation light and water molecules, leading to a significant amplification of this Raman signal. (2) The peak at 465 nm is identified as the intrinsic fluorescence characteristic peak of the PBAT microplastics. Its direct enhancement is due to the increased contribution from a greater quantity of the fluorescent material.

The fluorescence intensity of the suspension was further measured at these characteristic peaks at 436 nm and 465 nm, and the mass concentration–fluorescence intensity relationship of microplastics in ultrapure water was analyzed. The linear correlation analysis reveals distinct relationships between signal intensity and concentration across different ranges. At 436 nm, the characteristic peak shows a weak linear correlation (R^2^ = 0.425) across the full concentration range of 0–500 mg/L (Figure 4b). However, this correlation improves significantly within the narrower, higher concentration range of 100–500 mg/L, achieving an R^2^ of 0.877 (Figure 4c). In contrast, the intrinsic PBAT fluorescence peak at 465 nm only emerges above 100 mg/L. Within this 100–500 mg/L range (excluding the anomalous data point at 400 mg/L), the peak intensity demonstrated a highly linear positive correlation with concentration, with an R^2^ of 0.963. Unfortunately, the investigation of fluorescence at higher microplastic concentrations is precluded by the inability to form a homogeneous suspension.

Furthermore, in order to investigate the influence of the shape and size of microplastics on the constructed mass concentration–fluorescence intensity relationship model, 50 μm-PBAT microspheres with more uniform size (close to 60 μm after passing through 140–300 mesh sieve, Figure S6) were selected to prepare suspensions with the same concentration gradient, and the fluorescence was characterized. Similarly, characteristic peaks are observed in the fluorescence spectra at both 436 nm and within the 450–500 nm range, which generally intensifies with increasing concentration (Figure S7a). Unlike the previous findings, the signal at 436 nm maintains a strong linear correlation with mass concentration across a broad range of 0–500 mg/L (Figure S7b,c), indicating that the use of uniform microspheres can improve the characterization accuracy for low-concentration (<100 mg/L) microplastics. However, due to the broad profile of the intrinsic fluorescence peak of the PBAT microspheres, precise determination of its peak value is challenging.

#### 3.2.2. Comparison of Fluorescence Labeling and Intrinsic Fluorescence Quantitative Method

6-Aminofluorescein (AF) is commonly used to label the carboxyl group in polymers, and it has been proved that a good linear relationship (R^2^ = 0.99) can be obtained between the fluorescence increase and the concentration of carboxyl group [53]. Therefore, the carboxyl group in PBAT was labeled by AF to obtain fluorescently labeled PBAT particle microplastics (particle size of 140–300 mesh), and a series of suspensions with the same concentration gradient were prepared. Under the excitation of 492 nm, AF molecule shows a bright green emission at 518 nm (Figure 4g), which is consistent with the literature [53]. Therefore, under the excitation of 492 nm, it is observed that the fluorescence intensity of this series of suspensions increases almost with the increase of concentration at 518 nm. The linear correlation model between mass concentration and fluorescence intensity in the range of 0–500 mg/L is obtained, and the R^2^ reaches 0.934. This value is slightly higher than the R^2^ of 0.877 obtained from the scattering enhancement-based model but is comparable to the R^2^ of 0.963 achieved by the model based on the intrinsic fluorescence of PBAT microplastics. Although neither model reaches an R^2^ of 0.99, it should be noted that the fluorescence intensity emitted by the fluorescence labeling method is as high as 10^6^, whereas the intrinsic fluorescence intensity of microplastics is only 10^3^, indicating that the latter is more susceptible to environmental interference in complex settings.

It should be noted that this study aims to explore the feasibility of quantifying BMPs based on their intrinsic fluorescence, with the important premise that the microplastics have been isolated and purified from complex environmental matrices such as soil, seawater, or compost. The positioning of this method is to replace traditional techniques like visual counting for the rapid quantitative analysis of purified microplastics. In the standard analytical workflow, microplastics enriched on filter membranes can be repeatedly rinsed with ultrapure water, transferred, and re-suspended to prepare a suspension with an approximately known concentration range (above the 100 mg/L threshold), followed by fluorescence testing and accurate quantification. Since this workflow effectively removes the vast majority of environmental background substances through the purification step, the method, at this stage, can circumvent the fluorescence interference caused by complex environmental components.

To verify the potential impact of environmental background fluorescence, we further examined three liquid matrices commonly encountered in microplastic separation procedures: (1) filtered seawater; (2) saturated ZnCl_2_ solution, commonly used for density separation; and (3) the filtrate obtained from soil flotation using saturated ZnCl_2_ solution. As shown in Figure S8, compared to ultrapure water, all three matrices exhibited significant background fluorescence peaks within the 450–550 nm range. These results indicate that without adequate purification steps, such intense background signals would severely mask the weak fluorescence changes induced by the presence of BMPs, making direct in situ fluorescence quantification in complex mixtures unfeasible.

## 4. Conclusions

Biodegradable plastics solve the plastic problem from the source, but they are prone to disintegrate during degradation to produce microplastics, which has aroused people’s attention and concern. Therefore, it is urgent to establish an efficient and accurate quantitative detection method for microplastics. In this study, the shapes and sizes of different biodegradable polyester microplastics (PLA, PHB, PBS, PBAT, and PCL) and their fluorescence properties were systematically investigated; we successfully demonstrated the feasibility of a label-free quantitative method for biodegradable polyester microplastics (PBAT) by leveraging their intrinsic fluorescence.

All studied biodegradable polyester microplastics exhibited two types of intrinsic fluorescence emission: fluorescence originating from molecularly characterized functional groups and chromophore fluorescence formed by aggregation of conjugated groups. Microplastic fluorescence is significantly affected by size and shape: as the particle size decreases, the fluorescence intensity decreases and is accompanied by a redshift; film microplastics show a more pronounced decrease in fluorescence intensity and a redshift compared to particle microplastics.

Using PBAT as a model, under 380 nm excitation, two distinct concentration-dependent signals in the suspension are successfully resolved: the signal at 436 nm, originating from indirectly enhanced water Raman scattering by microplastics, and the intrinsic PBAT fluorescence peak at 465 nm. Based on this, quantitative models are established, with correlation R^2^ of 0.877 and 0.963, respectively, over 100–500 mg/L, laying a methodological foundation for accurate quantification.

Through a systematic comparison with the fluorescence labeling method, this study objectively evaluates the advantages and limitations of the intrinsic fluorescence method. Although it exhibits lower signal intensity and weaker resistance to interference in complex environments, it eliminates the need for cumbersome labeling procedures. This provides an irreplaceable advantage in terms of operational simplicity and analysis speed, making it particularly suitable for the rapid screening and quantification of pre-isolated and purified microplastics, providing a foundational new strategy for the development of rapid, non-destructive detection technologies.

## Figures and Tables

**Table 1:** Property parameters of PET and biodegradable polyester raw materials.

Sample	*M*_n_ [g mol^−1^]	*M*_W_ [g mol^−1^]	PI
PET	14,754	45,101	3.06
PLA	64,436	75,665	1.17
PHB	54,389	74,774	1.37
PBAT	31,447	56,278	1.79
PBS	47,739	67,853	1.42
PCL	70,384	75,958	1.08

**Table 2:** Average particle size parameters of PET and biodegradable polyester microplastics.

Sample	Mesh Size of Sieve					
	20–40		40–140		140–300	
	Particles	Fragments	Particles	Fragments	Particles	Fragments
PET	900 μm	-	190 μm	-	80 μm	-
PLA	760 μm	-	310 μm	-	120 μm	-
PHB	710 μm	-	160 μm	-	60 μm	-
PBAT	720 μm	410 μm	230 μm	250 μm	60 μm	120 μm
PBS	790 μm	-	180 μm	-	60 μm	-
PCL	780 μm	-	250 μm	-	60 μm	-

## Data Availability

Data is contained within the article or Supplementary Material.

## Supplementary Materials

The following supporting information can be downloaded at: https://www.mdpi.com/article/10.3390/polym17212953/s1, Figure S1: SEM distribution of PET and different biodegradable polyester microplastics: (a) PET (from pellets), (b) PLA (from pellets), (c) P(3HB-co-4HB) (from pellets), (d) PBAT (from pellets), (e) PBAT (from film), (f) PBS (from pellets), (g) PCL (from pellets); Numbers 1–3 represent microplastics with a mesh range of 20–40, 40–140 and 140–300; Figure S2: Particle size distribution of PET and different biodegradable polyester microplastics: (a) PET (from pellets), (b) PLA (from pellets), (c) P(3HB-co-4HB) (from pellets), (d) PBAT (from pellets), (e) PBAT (from film), (f) PBS (from pellets), (g) PCL (from pellets); Numbers 1–3 represent microplastics with a mesh range of 20–40, 40–140 and 140–300; Figure S3: Photographs of PET and different biodegradable polyester microplastics under daylight and 365 nm UV light: a mesh range of (a) 20–40; (b) 40–140; (c) 140–300; Figure S4: Fluorescence spectra of PET and different biodegradable polyester microplastics under 365 nm UV excitation. Figure S5: Fluorescence emission spectra of different PBAT microplastic sizes: (a) PBAT film microplastics at 270 nm and 380 nm excitation wavelengths; (b) PBAT film microplastics at 365 nm excitation wavelength; (c) PBAT particle and film microplastics at 365 nm excitation wavelength; Figure S6: (a) SEM distribution of PBAT microspheres; (b) Size distribution of PBAT microspheres. Figure S7 (a) Fluorescence spectra of different concentrations of 50-μm PBAT microspheres suspensions under excitation at 380 nm; (b) and (c) are the mass concentration-fluorescence intensity linear fitting curve of (a) at 436 nm. Figure S8 Fluorescence spectra of common aqueous solution environment at 380 nm excitation wavelength.
