# Supplementary material for: Feasibility Study on Quantification of Biodegradable Polyester Microplastics Based on Intrinsic Fluorescence
Source: Polymers (Basel). 2025 Nov 5;17(21):2953. doi: 10.3390/polym17212953 (PMC12609927; doi:10.3390/polym17212953)
Supplement: Supplementary file 1 [file polymers-17-02953-s001.zip › polymers-3935077-supplementary.pdf]

Supplemental Information

Feasibility study on quantification of biodegradable polyester microplastics based on intrinsic fluorescence

Tian-Chao Shi[1,2], Ze-Yang Zhang[2], Xiao-Han Zhou[1,2], Xing Zhang[1], Shao-Chuang Su[1], Hong Yang[1], Hao-Bo Chai[1], Ge-Xia Wang[2], Jun-Hui Ji[2], Yue Ding [3*], Xu-Ran Liu[1*] and Dan Huang[2*]

[1] College of Materials Engineering, North China Institute of Aerospace Engineering, Langfang Hebei 065000, China;

[2] National Engineering Research Center of Engineering and Ecological Plastics, Technical Institute of Physics and Chemistry, Chinese Academy of Sciences, Beijing 100190, China;

[3] School of Material and Chemical Engineering, Xuzhou University of Technology, Xuzhou 221018, China.

Corresponding author:

dingyue@xzit.edu.cn; liuxuran15@mails.ucas.ac.cn;

danhuang@mail.ipc.ac.cn

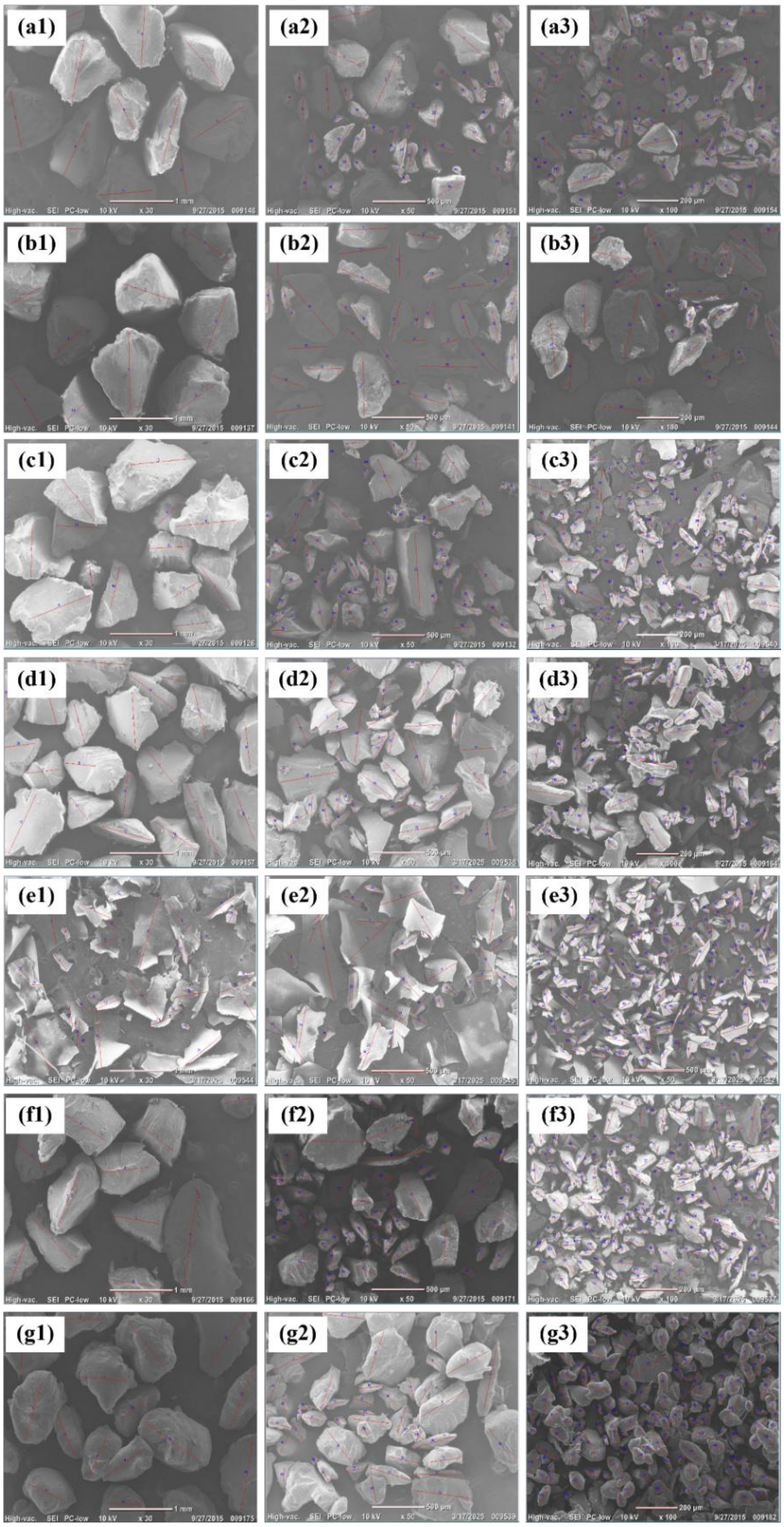

Figure S1 SEM distribution of PET and different biodegradable polyester microplastics: (a) PET (from pellets), (b) PLA (from pellets), (c) P(3HB-co-4HB) (from pellets), (d) PBAT (from pellets), (e) PBAT (from film), (f) PBS (from pellets), (g) PCL (from pellets); Numbers 1-3 represent microplastics with a mesh range of 20-40, 40-140 and 140-300.

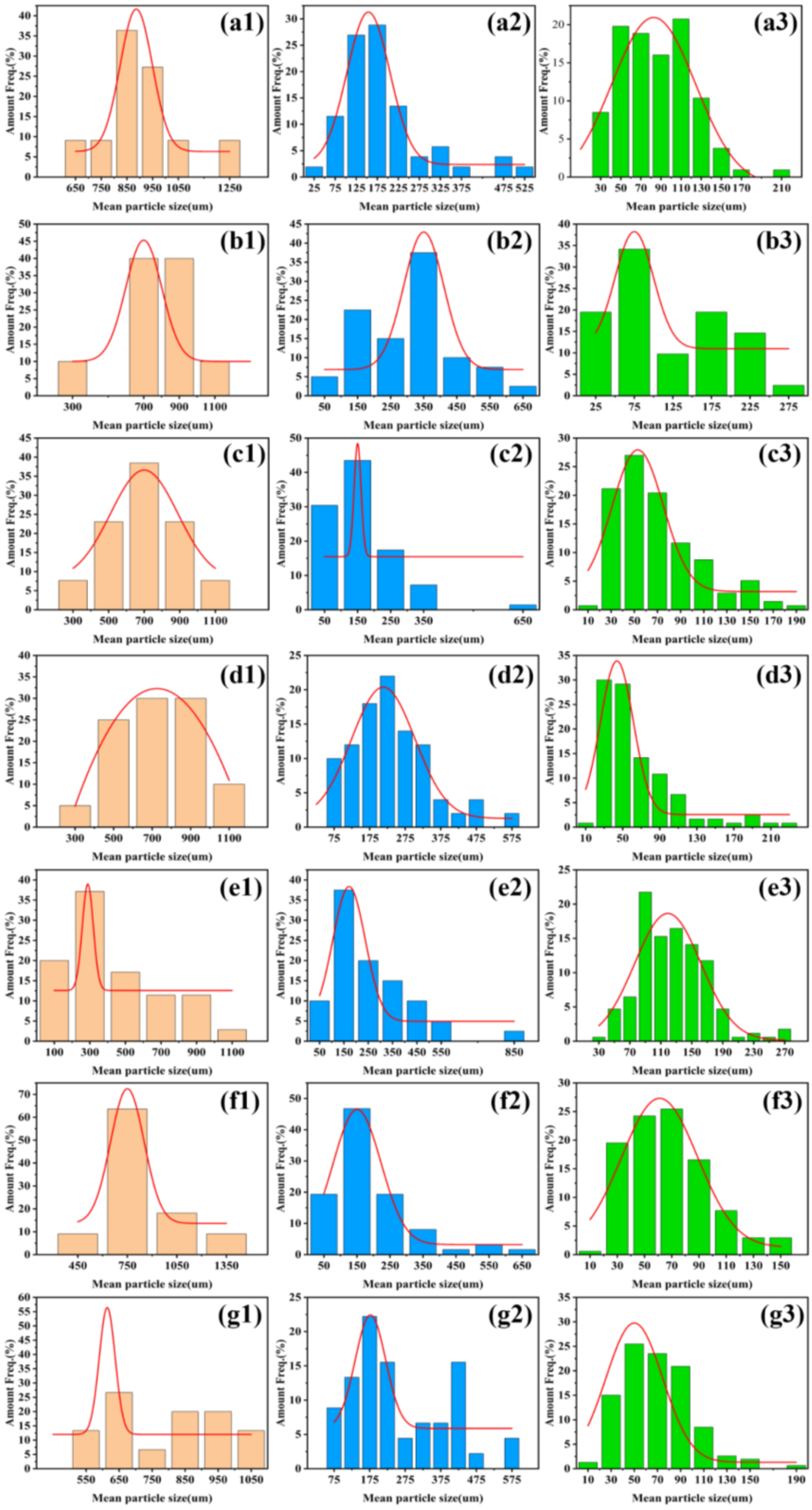

Figure S2 Particle size distribution of PET and different biodegradable polyester microplastics: (a) PET (from pellets), (b) PLA (from pellets), (c) P(3HB-co-4HB) (from pellets), (d) PBAT (from pellets), (e) PBAT (from film), (f) PBS (from pellets), (g) PCL (from pellets); Numbers 1-3 represent microplastics with a mesh range of 20-40, 40-140 and 140-300.

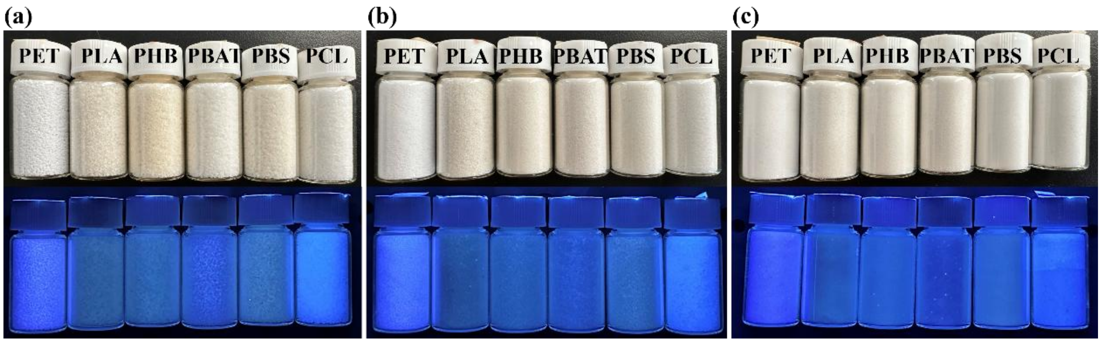

Figure S3 Photographs of PET and different biodegradable polyester microplastics under daylight and 365 nm UV light: a mesh range of (a) 20-40; (b) 40-140; (c) 140-300.

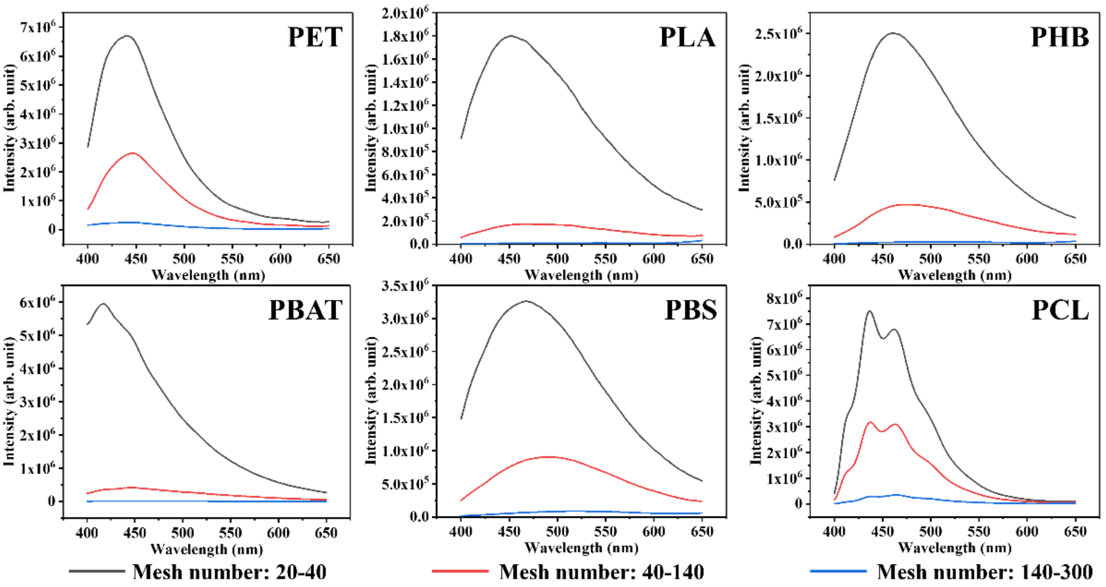

Figure S4 Fluorescence spectra of PET and different biodegradable polyester microplastics under 365 nm UV excitation.

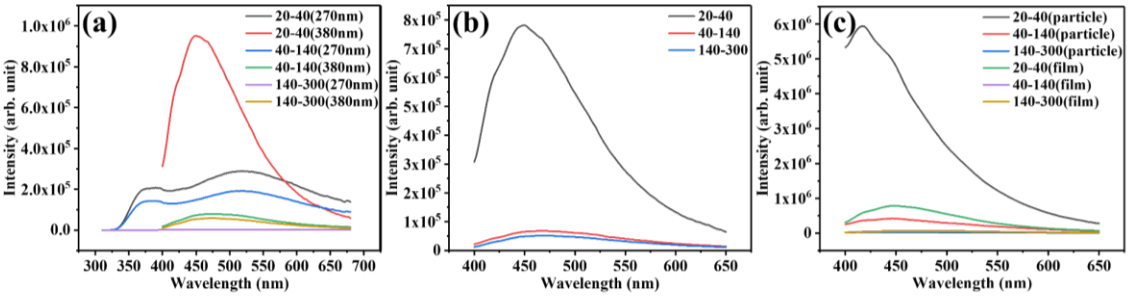

Figure S5 Fluorescence emission spectra of different PBAT microplastic sizes: (a) PBAT film microplastics at 270 nm and 380 nm excitation wavelengths; (b) PBAT film microplastics at 365 nm excitation wavelength; (c) PBAT particle and film microplastics at 365 nm

excitation wavelength.

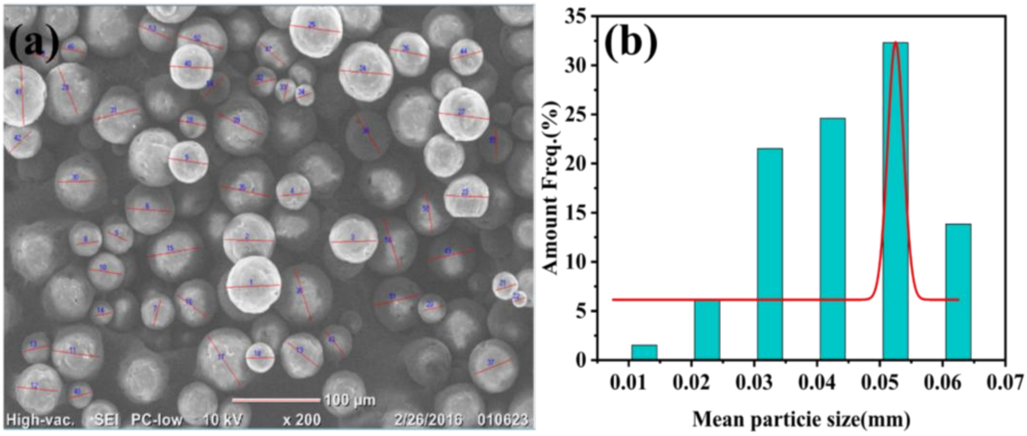

Figure S6 (a) SEM distribution of PBAT microspheres; (b) Size distribution of PBAT microspheres.

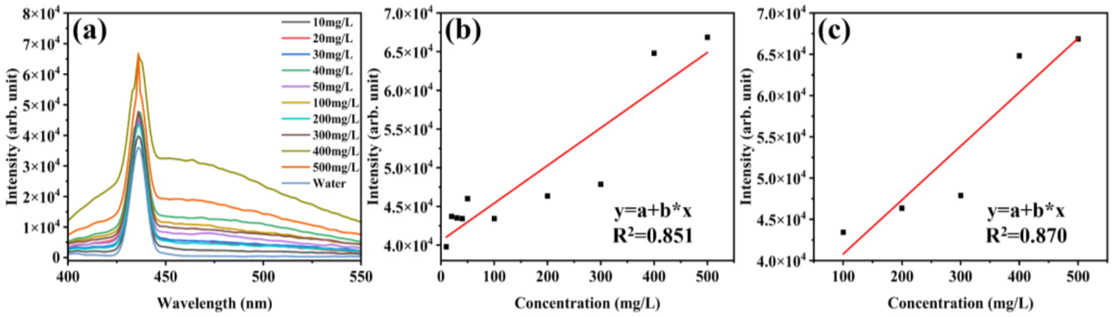

Figure S7 (a) Fluorescence spectra of different concentrations of 50-μm PBAT microspheres suspensions under excitation at 380 nm; (b) and (c) are the mass concentration-fluorescence intensity linear fitting curve of (a) at 436 nm.

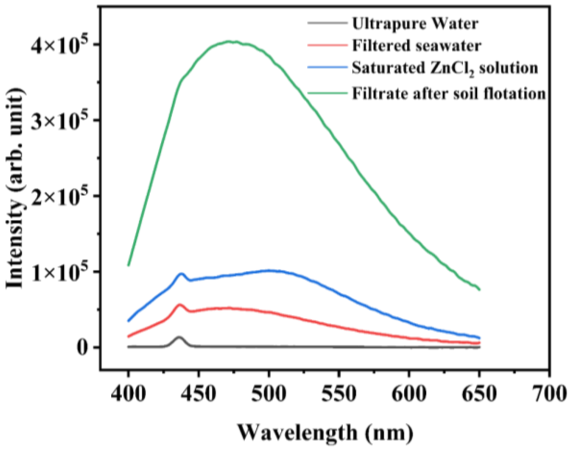

Figure S8 Fluorescence spectra of common aqueous solution environment at 380 nm excitation wavelength.
